# Physical activity interventions to improve daily walking activity in cancer survivors

**DOI:** 10.1186/1471-2407-10-406

**Published:** 2010-08-04

**Authors:** Ruud H Knols, Eling D de Bruin, Kei Shirato, Daniel Uebelhart, Neil K Aaronson

**Affiliations:** 1 Department of Rheumatology and Institute of Physical Medicine, University Hospital Zurich, Switzerland; 2 Institute of Human Movement Sciences and Sport, ETH Zurich, Zurich, Switzerland; 3 Division of Psychosocial Research and Epidemiology, The Netherlands Cancer Institute, Amsterdam, The Netherlands

## Abstract

**Background:**

Cancer patients may benefit from physical exercise programs. It is unclear, however, how sustained levels of physical activity are best achieved in this population. A systematic review was performed to summarize the current evidence of the effect of physical activity interventions on daily walking activity enhancement in cancer survivors, and to review the literature for its methodological quality.

**Methods:**

A search in Medline, PEDro and the Cochrane databases was performed for English literature citations (randomized controlled trials; 'RCTs'). In a first step, one reviewer abstracted data from the included studies on patients, physical activity interventions and outcomes. Two independent reviewers reviewed the methodological quality of these studies. Data were pooled using random-effects calculations.

**Results:**

Our search identified 201 citations. Five RCTs that reported changes in daily step activity over time were identified, and were reviewed for methodological quality and substantive results. The median score across studies for methodological quality based on the PEDro criteria was 8. These 5 RCTs evaluated 660 participants with a mean age of 53.6 (SD 4.2) years. The mean change in daily step activity for patients with a physical exercise intervention was 526 daily steps (SD 537), with a range from -92 to 1299 daily steps. The data of three studies reporting the effect of combined physical activity and counseling on daily walking activity in breast cancer survivors were pooled, however; the I^2 ^was 79%, indicating statistical heterogeneity between the three trials.

**Conclusion:**

The 5 RCTs reviewed were of good methodological quality. Together they suggest that combined physical activity and counseling improves daily step activity in (breast) cancer survivors. Studies that define a step goal appear to be more effective in improving daily walking activity than studies that do not do so. However, the current results should be interpreted with caution because of the observed clinical and statistical heterogeneity. Future studies are warranted to evaluate the effects of goal targeted physical activity, with or without counseling, on daily walking in various cancer populations.

## Background

Cancer is increasingly being viewed as a chronic illness requiring long-term management, and thus there is a growing need for evidence-based rehabilitation interventions for cancer survivors [1]. Physical exercise programs have been developed with the aim of improving a range of outcomes, including physical performance, body composition, hemoglobin concentration, immune function, fatigue, psychological well-being and health-related quality-of-life (HRQOL) [2-4]. There is increasing evidence that cancer patients (e.g., breast, colon, and prostate cancer, hematological malignancies) [5,6] may benefit from physical exercise programs in terms of improvement in fitness levels, physical activity and HRQOL [7].

Walking is a major component of daily physical activity, and is the most common form of exercise [8]. Walking is self regulated in intensity, duration and frequency, and can be an important indicator of a person's health and fitness status [9]. Among healthy individuals, 10,000 steps daily have been estimated to be of value in maintaining desired health benefits [10]. For cancer survivors, however, it is unclear whether an increase in walking activity results in enhanced functional health.

Improved functional status is a primary goal in the rehabilitation of cancer survivors [11]. Relatively new techniques allowing unobtrusive long-term activity monitoring (e.g., with the use of pedometers or microprocessor-based accelerometer recorders) may provide a clearer indication of how much an individual actually walks in daily life [12]. Two reports indicate that the use of activity monitors may significantly increase physical activity levels among children and adolescents [13], as well as in adults [14] with and without chronic health conditions. One report described expected values for daily walking activity in breast cancer patients, and hypothesized that such activity monitoring may facilitate physical activity in this population [15]. Although the evidence on the effect of physical activity interventions on daily step activity in cancer survivors has not been established, activity monitors have recently experienced a surge in popularity as a tool for motivating patients and monitoring physical activity [14,16].

We conducted a systematic review to summarize the currently available evidence from randomized clinical trials (RCT's) on the effect of physical activity interventions on quantified daily walking behavior in cancer survivors. The secondary aim was to evaluate the methodological quality of these studies.

## Methods

### Data sources and search strategies

Individualized search strategies for the Medline, Cochrane and PEDro databases were developed in collaboration with a librarian from the *Eidgenössisch Technische Hochschule *(ETH), Zurich. We used medical sub-headings as search terms, including behavior, cancer, neoplasm, chronic disease, monitoring, motor activity, physical activity interventions, physical exercise, physical therapy modalities, physical therapy techniques, yoga, random* or random allocation and the free text words accelerometer, pedometer, step counter, daily steps, physical activity and concealed. We also reviewed the bibliographies of retrieved articles and relevant conference proceedings. Searches were performed in all databases up to November 2009.

### Study selection

A study was considered eligible for inclusion in the review when it was a RCT, examining the results of a physical activity intervention on daily walking activity in cancer patients. Physical activity interventions were defined as walking, physical exercise, counseling, yoga, relaxation or a combination of these. Studies that evaluated the effectiveness of drugs, nutrition, transcutaneous electrical stimulation and mineral or vitamin supplements were excluded from this review. Studies had to have made use of pedometers, step counters or accelerometers that monitored daily walking activity. We excluded studies of hospitalized patients. The review was limited to English language publications.

### Data extraction

#### Methodological quality

Two reviewers (EDB and KS) independently assessed the methodological quality of the studies according to the PEDro scale. The PEDro scale is based on the Delphi list developed by Verhagen et al. [17], which is a set of 11 criteria for quality assessment: 1) eligibility; 2) use of randomization; 3) concealment of treatment allocation; 4) equivalence (or similarity) of groups at baseline; 5) blinding of the subjects; 6) blinding of the therapists, 7) blinding of the outcome assessors; 8) intention-to-treat analyses; 9) reporting of point estimates; 10) measures of variability of the primary outcome and 11) adequacy of follow-up and use of between-group statistical comparisons [18]. Two of the three criteria relating to the use of blinding procedures were not rated because it is difficult, if not impossible, to blind patients and care providers to treatment assignment in this area of research [19]. Thus, nine of the eleven quality criteria were evaluated in this systematic review. For each quality criterion, three rating categories were available: "yes, met criteria"; "no, did not meet criteria"; and "do not know."

Percentage agreement and Cohen's kappa were calculated with GRAPHPAD software (Version 2002-2005; GRAPHPAD Software Inc, San Diego, Ca), and were interpreted in accordance with Landis and Koch's benchmarks for assessing the agreement between raters: poor (<0), slight (0.0 to 0.20), fair (0.21 to 0.40), moderate (0.41 to 0.60), substantial (0.61 to 0.80), and almost perfect (0.81 to 1.0) [20]. Disagreement regarding inclusion of the studies was resolved by consensus between authors (RHK, EDB, KS). As recommended by van Tulder et al., [21] a third reviewer (DU) was used in the event of any disagreement between the two reviewers regarding the methodological quality of a trial. The PRISMA-statement was followed for reporting items of this systematic review and meta-analyses [22].

#### Content of the studies

One author (RHK) independently abstracted the following information from each of the studies included in this review: 1) design and sample; 2) inclusion criteria; 3) type of intervention; 4) type of walking activity monitoring system; 5) change in steps per day; and 6) conclusions from the studies and statistical significance. In case a study reported both immediate post-intervention and follow-up data, we reported the post-intervention data.

#### Data synthesis and analysis

The study results were pooled, if appropriate, using a random effects model after evaluating heterogeneity. Heterogeneity of the study findings was assessed with the I^2 ^statistic, where a value greater than 50% is considered to indicate substantial heterogeneity [23]. For continuous daily step outcomes, the effect-size and its 95%CI were reported when data were on a uniform scale. For the summary effect size, we assessed statistical heterogeneity by calculating the Q-statistic. An effect size with a two-sided probability of less than 0.05 was deemed to be heterogeneous. Publication bias was tested using the fail-safe N, which signifies the number of studies that would be required to change a significant summary effect to one that was not statistically significant [14]. Comprehensive Meta-analyses software (BIOSTAT, Englewood NJ, USA) was used for the analyses of pooled data.

## Results

**Figure 1:**
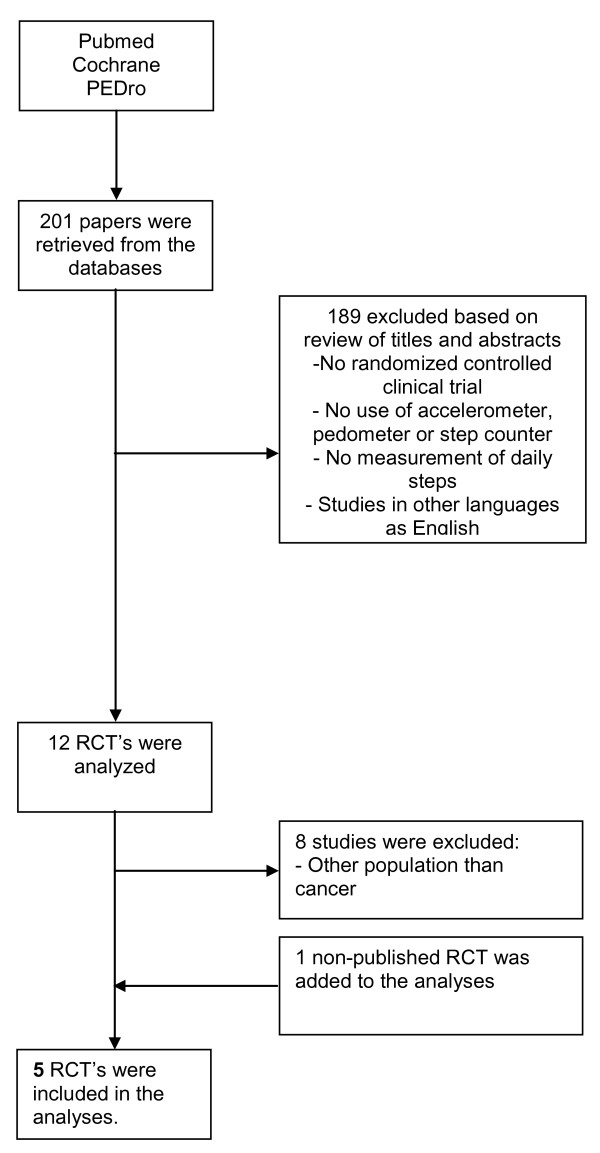
**Flowchart of the systematic review**.

Our search identified 201 potentially relevant articles (Figure 1). Four published RCT's [24-27] and one RCT [Knols RH, de Bruin ED, Uebelhart D, Aufdemkampe G, Schanz U, Stenner-Liewen F, Hitz F, Taverna C, Aaronson NK: Effects of an outpatient physical exercise program in hematopoietic stem-cell transplantation recipients: a randomized clinical trial, unpublished] that was submitted for publication at the time of inquiry, met the eligibility criteria and were included in the final analysis. We identified three [24,29,30] reports from the one Yale Exercise and Survivorship study. Although these three papers focused on different research questions [24,29,30] they essentially were all based on the same set of data for the parameters of interest for this review. Therefore, they were treated as one publication. The report of Irwin et al. [24] was used in this systematic review, as this paper provided means and standard deviations of pedometer steps/day for baseline and post-treatment, that were needed for the meta-analysis.

### Methodological quality

The results of the methodological quality assessment are presented in table 1. The reviewers agreed on 44 of 45 methodological ratings (97.8%). The remaining disagreements were resolved after discussions among the reviewers. The inter-reviewer κ statistic was 0.79 (95% CI, 0.38 to 1.20). The median criteria score on the Delphi list (range 1 to 9) was 8 (Table 1). The studies of Irwin et al. [24] and Knols et al. [Knols RH, de Bruin ED, Uebelhart D, Aufdemkampe G, Schanz U, Stenner-Liewen F, Hitz F, Taverna C, Aaronson NK: Effects of an outpatient physical exercise program in hematopoietic stem-cell transplantation recipients: a randomized clinical trial, unpublished] were rated positively on all nine methodological criteria.

**Table 1:** Methodological quality of the included studies

Item→ ↓Study	Eligibility	Randomly allocated	Concealed allocation	Similar at baseline	Blinding of assessors	Measures from more than 85%	Intention-to-treat	Between group comparison	Point-estimates and measures of variability	Total items reported
**Irwin**[24]	+	+	+	+	+	+	+	+	+	9

Rogers [25]	+	+	+	+	?	+	+	+	+	8

Vallence [26]	+	+	+	+	?	+	+	+	+	8

Matthews [27]	+	+	-	+	-	+	+	+	+	7

[Knols RH, de Bruin ED, Uebelhart D, Aufdemkampe G, Schanz U, Stenner-Liewen F, Hitz F, Taverna C, Aaronson NK. Effects of an outpatient physical exercise program in hematopoietic stem-cell transplantation recipients: a randomized clinical trial, Unpublished]	+	+	+	+	+	+	+	+	+	9

Total items	5	5	4	5	2	5	5	5	5	

+; met criteria on PEDro-scale, -; did not met criteria on PEDro-scale, ?; don't know.

All 5 studies [[24-27] & Knols RH, de Bruin ED, Uebelhart D, Aufdemkampe G, Schanz U, Stenner-Liewen F, Hitz F, Taverna C, Aaronson NK. Effects of an outpatient physical exercise program in hematopoietic stem-cell transplantation recipients: a randomized clinical trial, unpublished] explicitly stated the eligibility criteria employed, reported using an appropriate method to generate the random allocation sequence, reported group similarity at baseline for the most important prognostic indicators, were successful in obtaining at least 85% of the data for the primary outcome(s), performed an intention-to-treat analysis, provided between group comparisons and provided point estimates and measures of variability for the primary outcome(s). Four of 5 studies reported using an appropriate method to generate concealment of allocation [[24-26] & Knols RH, de Bruin ED, Uebelhart D, Aufdemkampe G, Schanz U, Stenner-Liewen F, Hitz F, Taverna C, Aaronson NK. Effects of an outpatient physical exercise program in hematopoietic stem-cell transplantation recipients: a randomized clinical trial, unpublished]. The outcome assessors were blinded in 2 of 5 studies [[24] & Knols RH, de Bruin ED, Uebelhart D, Aufdemkampe G, Schanz U, Stenner-Liewen F, Hitz F, Taverna C, Aaronson NK. Effects of an outpatient physical exercise program in hematopoietic stem-cell transplantation recipients: a randomized clinical trial, unpublished].

### Study characteristics

In all studies, the participants wore the activity monitors for 7 consecutive days [[24-27] & Knols RH, de Bruin ED, Uebelhart D, Aufdemkampe G, Schanz U, Stenner-Liewen F, Hitz F, Taverna C, Aaronson NK: Effects of an outpatient physical exercise program in hematopoietic stem-cell transplantation recipients: a randomized clinical trial, unpublished]. One study reported measurements for 7 days, but included 4 valid days for assessment [25].

Different instruments for measuring physical activity were used across the studies: an Actigraph MTI model 71256 [27], a GT1 M Actigraph accelerometer [25], a Cymatech step activity accelerometer SAM3 [Knols RH, de Bruin ED, Uebelhart D, Aufdemkampe G, Schanz U, Stenner-Liewen F, Hitz F, Taverna C, Aaronson NK: Effects of an outpatient physical exercise program in hematopoietic stem-cell transplantation recipients: a randomized clinical trial, unpublished], and a New lifestyles digi-walker SW-200 [26]. One trial did not report the type accelerometer or pedometer used [24].

The drop-out rate between baseline and post-treatment varied between 0% [27] and 13% [Knols RH, de Bruin ED, Uebelhart D, Aufdemkampe G, Schanz U, Stenner-Liewen F, Hitz F, Taverna C, Aaronson NK: Effects of an outpatient physical exercise program in hematopoietic stem-cell transplantation recipients: a randomized clinical trial, unpublished]. Two studies [24,26] used non-sealed pedometers and instructed the participants to record the daily steps taken at the end of each day of the measurement period. One study used a sealed accelerometer [Knols RH, de Bruin ED, Uebelhart D, Aufdemkampe G, Schanz U, Stenner-Liewen F, Hitz F, Taverna C, Aaronson NK. Effects of an outpatient physical exercise program in hematopoietic stem-cell transplantation recipients: a randomized clinical trial, unpublished].

Two studies [24,26] included a step diary in the activity program and 4 trials included physical activity counseling, [24-27] with a median of 4 counseling sessions (range 4-8 sessions). Three studies were performed in the United States [24,25,27], 1 in Canada [26], and 1 in Switzerland [Knols RH, de Bruin ED, Uebelhart D, Aufdemkampe G, Schanz U, Stenner-Liewen F, Hitz F, Taverna C, Aaronson NK: Effects of an outpatient physical exercise program in hematopoietic stem-cell transplantation recipients: a randomized clinical trial, unpublished].

### Patient characteristics

In total, 660 participants were included in the studies reviewed. Their mean age (SD) was 53.6 (SD 4.2) years. All studies included participants older than 60 years [[24-27] & Knols RH, de Bruin ED, Uebelhart D, Aufdemkampe G, Schanz U, Stenner-Liewen F, Hitz F, Taverna C, Aaronson NK: Effects of an outpatient physical exercise program in hematopoietic stem-cell transplantation recipients: a randomized clinical trial, unpublished].

Across studies, only 11.7% of the sample was male. Four studies were on breast cancer and enrolled women only [24-27]. One study was with hematopoietic stem cell transplantation recipients [Knols RH, de Bruin ED, Uebelhart D, Aufdemkampe G, Schanz U, Stenner-Liewen F, Hitz F, Taverna C, Aaronson NK: Effects of an outpatient physical exercise program in hematopoietic stem-cell transplantation recipients: a randomized clinical trial, unpublished].

Four studies reported participants' race/ethnicity [24-27]. Across these studies, on average, 71% was white (SD 32%). All studies reported participants' education [[24-27] & Knols RH, de Bruin ED, Uebelhart D, Aufdemkampe G, Schanz U, Stenner-Liewen F, Hitz F, Taverna C, Aaronson NK: Effects of an outpatient physical exercise program in hematopoietic stem-cell transplantation recipients: a randomized clinical trial, unpublished]. The mean (SD) percentage of participants with a college degree or higher was 38% (SD 15%). Three studies reported the time since diagnosis [24,26,27]. The median time since diagnosis was 2.6 years (SD 1.2 years, range 0.8-3.3 years). Four studies reported body mass index [[24,26,27], Knols RH, de Bruin ED, Uebelhart D, Aufdemkampe G, Schanz U, Stenner-Liewen F, Hitz F, Taverna C, Aaronson NK: Effects of an outpatient physical exercise program in hematopoietic stem-cell transplantation recipients: a randomized clinical trial, unpublished]: the mean BMI percentage was 27.6% (SD 3.0%).

One report described obesity of the participants; 111 of the 377 participants had a BMI >30 (29.4%); 77 of these 111 patients (20.4%) had a BMI between 30.0 and 34.9, 17 (4.5%) patients had a BMI between 35.0 and 39.9 and 17 patients had a BMI ≥40 [26]. Two studies reported participants to be physically active at baseline. One study reported that one third of the participants performed physical exercise [26], and the other study reported that two-thirds of the participants were physically active for at least 20 minutes per day [24]. On average, across all studies, participants made 6285 steps (SD 1344) per day at baseline, with a range from 4697 to 8178. The mean change in daily step activity for patients with a physical exercise intervention was 527 daily steps (SD 536, with a range from -92 to 1299 daily steps.

A variety of physical activity modalities were employed: combined supervised endurance training (swimming, aerobics, other forms or a combination) plus individual exercise at a health club [24], walking [25,27], behavior change intervention including discussion groups [25], supervised and home exercise [24,25], face-to-face counseling [25], telephone counseling [26,27], physical activity motivators as a standard public health recommendation [26], a leaflet with information about physical activity [25,26], pedometers [24,26], combined supervised resistance strength and endurance training [Knols RH, de Bruin ED, Uebelhart D, Aufdemkampe G, Schanz U, Stenner-Liewen F, Hitz F, Taverna C, Aaronson NK: Effects of an outpatient physical exercise program in hematopoietic stem-cell transplantation recipients: a randomized clinical trial, unpublished], or combinations of the above (see table 2). Two RCTs reported the intensity of the physical exercise interventions. One trail used an intensity of 60-80% of the maximum heart rate [24] and one RCT offered exercise up to 75% of the maximum heart rate [Knols RH, de Bruin ED, Uebelhart D, Aufdemkampe G, Schanz U, Stenner-Liewen F, Hitz F, Taverna C, Aaronson NK: Effects of an outpatient physical exercise program in hematopoietic stem-cell transplantation recipients: a randomized clinical trial, unpublished]. In one RCT, in the first four weeks, the goal was to walk three times/week (20-30 minutes/session); during week 5-7 to walk four times/week (30-40 minutes/session), and for the final 5 weeks of the study to walk 5 times/week (30-40 minutes/session) [27]. In one RCT they were advised to perform 30 minutes of moderate walking 5 times a week [25], and in one RCT the patients were advised to perform 30 minutes of vigorous walking 5 times a week [26]. The mean duration of the physical activity programs was 14.4 weeks (SD 5.4), ranging from 12 to 24 weeks.

**Table 2:** Effectiveness of physical exercise on physical activity assessed with wearable systems

Author [Ref]	Design and sample	Inclusion criteria	Type of intervention	Type of walking activity monitoring system	Change in steps per day	Statistical significances
Irwin [24]	RCT of PE (n = 37) or UC (n = 38).	Breast cancer survivors completed adjuvant treatment at least 6 months for enrolment in study, between 40 and 75 years, stage I-IIIA, less than 90 min./wk. moderate or vigorous activity levels, not exercising in weight-loss intervention, non- smoking, non-diabetic or non-history of invasive cancer.	Combined supervised training intervention, 3x/wk, 60-80% at supervised health-club, with 2-5 participants and individual exercise at home. (2x/wk, 30 min).	Steps/day assessed with a 7-day pedometer log. Patients wore the pedometer from awakening in the morning until they went to bed in the evening.	PE group increased pedometer steps by 1621/day, compared to a decrease of 60 steps/day for the UC group.	*p *< 0.01/*d *= 0.38.

Rogers [25]	RCT of 12 week behavioral change activity group (n = 21) and UC group (n = 20) with measurements at baseline and post-intervention.	English speaking breast cancer survivors, stage I-IIIA, taking aromatase inhibitors or hormonal therapy for the duration of the study. Patients after surgery were delayed for 8 weeks before entering the study.	12-wk behavior change intervention contained discussion group sessions, supervised PE, home-based PE and update face-to-face counseling. The UC group contained written materials from the ACS.	Steps/day assessed with Actigraph accelerometer Type GT1 M for 7 consecutive days. 4 valid days were used for analyses.	Both the PE and UC group improved for total activity step counts. However, there was no statistical difference between both groups.	*p *< 0.81/*d *= 1.02.

Vallence [26]	RCT of SR (n = 96), PM (n = 94), PED (n = 94), COM (n = 93).	Breast cancer survivors after adjuvant chemotherapy, stage I-IIIA, not chronic medical and orthopaedic conditions, sufficient ability to read English, absence of breast cancer, not exercising in other programs.	All groups received SR, via telephone (total 5x/wk, 30 min). PM received a copy an exercise guide for breast cancer survivors, PED received pedometer and COM received both the pedometer and the copy of 'Exercise for health' by post after randomization.	Steps/day assessed with Digi-walker SW-200. Survivors in the COM and PED group (those who wore a step meter, n = 187) recorded the steps on 83.% (70 of 84 study days).	No differences between any of the groups for steps/day.	SR vs COM *p *= 0.710/*d *= 0.08. SR vs PED *p *= 0.885/*d *= 0.09. SR vs PM *p *= 0.727/*d *= 0.02. PED vs COM *p *= 0.848/*d *= 0.185. PM vs COM *p *= 0.982/*d *= 0.10.

Matthews 2006 [27]	RCT of PE (n = 23) and waiting list controls (n = 13) with assessments at 6 and 12 weeks.	Breast cancer survivors after adjuvant chemotherapy, stage I-IIIA, non-cardiovascular and orthopaedic conditions, non-exercising already more than 5 days/wk.	PE group 12 wk. walking program, UC received pedometer and counselling by telephone 30 min at start + 5 short telephone calls in wk. 1, 2, 4, 7 and 10 (10-15 min.) discussing safety, adherence, goal achievement, reinforcement and encouragement.	Steps/day assessed with Actigraph during 7 consecutive days.	Steps/day increased with 1153 steps in the PE group, but decreased in the control group with 559 steps.	*p *< 0.04/*d *= 0.82.

Knols RH, de Bruin ED, Uebelhart D, Aufdemkampe G, Schanz U, Stenner-Liewen F, Hitz F, Taverna C, Aaronson NK. Effects of an outpatient physical exercise program in hematopoietic stem-cell transplantation recipients: a randomized clinical trial, unpublished.	RCT of a 12 week supervised physical exercise program (n = 64) compared to UC (n = 67).	Adult patients with HSCT, GVHD except for grade I not requiring treatment, instable osteolyses, chronic pain, lesions of the central or peripheral nervous system, uncontrolled coronary pulmonary or diabetic disease.	PE group: 12- week supervised outpatient strength and endurance program, 2x/wk, 60-90 min. up to 75% of V02 _max _UC: usual care.	Steps/day assessed with SAM3 accelerometer for 7 consecutive days.	No differences for walking activity between the PE and the UC group.	*p *= 0.530/*d *= -0.04.

PE; Physical exercisers, UC; Usual care control group, ACS; American Cancer Society, SR; standard public-health recommendation, PM; Physical activity print materials, PED; step pedometer, COM; combination of PM and PED, HSCT; Hematopoietic stem cell transplantation, GVHD; Graft versus host disease, p; significance, d; effect-size.

### Effects of physical activity interventions on daily walking

Two studies yielded statistically significant results for change in daily step counts [24,27]. In these studies [24,27], a part of the program was helping patients to define a step target. Three studies did not achieve statistically significant changes for daily step counts [[25,26], Knols RH, de Bruin ED, Uebelhart D, Aufdemkampe G, Schanz U, Stenner-Liewen F, Hitz F, Taverna C, Aaronson NK: Effects of an outpatient physical exercise program in hematopoietic stem-cell transplantation recipients: a randomized clinical trial, unpublished]. A daily step goal definition was used in only one of these latter studies (table 2) [25]. Two reports from the Yale Exercise and Survivorship study [28,29] provided additional information on the outcome of interest. The participants in the physical exercise group increased their pedometer steps/week, on average by 1621 steps (11,347 steps/week [28] or approximately 0.9 miles/day [28,29] = 6.3 miles/week [28]), compared to 85 steps (595 steps/week or approximately 0.05 miles/day [28,29] (0.35 miles/week) [28] in the usual care group (p = 0.03) [28].

### Meta-analyses

Three RCTs were similar regarding patients, the interventions, and outcomes measures. All three of these studies were with breast cancer patients after primary medical treatment had been completed. All three studies investigated the combined effect of physical activity, counseling and goal setting, and used a usual care control group [24,25,27]. Across these studies, 152 patients were evaluated (n = 81 and 71 in the intervention and control groups, respectively). Their mean age was 54.4 (SD 2.4) years. All three studies included participants older than 60 years. The participants mean daily step average was 6377 steps (SD 822) at baseline, with a range from 5083 to 7409 steps. The duration of the intervention varied between 6 weeks [25] and 6 months [24] for supervised physical activity, and between six weeks of supervised and 10 week home-based activity in another trial [27]. One trial [25] included 10 weeks of home-based activity after the supervised program had been completed. All RCT's offered counseling as part of the program and used a pedometer which was carried at the waist. The patients were asked to record their daily steps to check if they achieved their target goals [24,25,27]. The mean change in daily step activity was 1099 daily steps (SD 2136), with a range from 1087 to 3182 steps.

**Figure 2:**
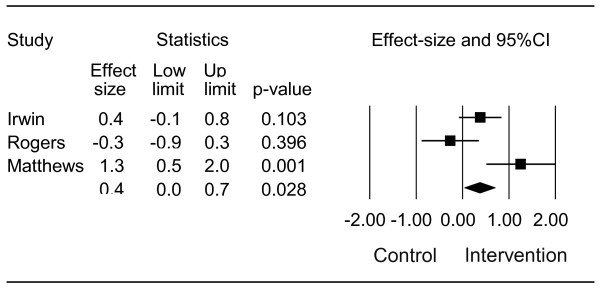
**Difference in effect-size of daily walking activity in breast cancer patients assigned to a physical activity intervention with step goal definition versus a control group: q-value = 9.508, df(Q) = 2, *p *= 0.009, I^2 ^= 79%**.

Statistical heterogeneity between the three trials was observed in meta-analyses after pooling; q-value = 9.508, df(Q) = 2, *p *= 0.009, I^2 ^= 79% (figure 2). The effect-size (ES) for the three studies combined was 0.4 (95%CI: lower limit 0.0, upper limit 0.7, *p *= 0.028). The evaluation of potential publication bias indicated that 2 missing studies would increase the *p*-value to greater than 0.05.

## Discussion

In this systematic review we evaluated the methodological quality and summarized the substantive results of studies of physical activity interventions for cancer patients designed to increase the level of daily walked steps. Five randomized clinical trials (RCTs) were identified, one satisfied 7 of 9 methodological quality criteria [27], two 8 of 9 criteria [25,26], and two all 9 quality criteria [[24], Knols RH, de Bruin ED, Uebelhart D, Aufdemkampe G, Schanz U, Stenner-Liewen F, Hitz F, Taverna C, Aaronson NK: Effects of an outpatient physical exercise program in hematopoietic stem-cell transplantation recipients: a randomized clinical trial, unpublished]. The most commonly observed problems were the blinding of the assessors and failure to report concealment of allocation (see table 2).

Together the results of these studies suggest that daily walking activity is likely to improve only when a realistic step goal is defined. Two of 5 reports that described a step goal were effective in improving daily step activity [24,27]. Conversely, the studies that did not define a step goal were not found to be effective in improving daily step activity [[26], Knols RH, de Bruin ED, Uebelhart D, Aufdemkampe G, Schanz U, Stenner-Liewen F, Hitz F, Taverna C, Aaronson NK: Effects of an outpatient physical exercise program in hematopoietic stem-cell transplantation recipients: a randomized clinical trial, unpublished].

The pooled results of the three studies examining the effect of physical activity on daily step activity showed a moderate effect (mean ES = 0.4). A statistically significant difference favouring the implemented exercise intervention was reported in two of three trials; however, the I^2 ^value was larger than 50%, indicating statistical heterogeneity between the pooled data. Moreover, a publication bias may have influenced the results of the meta-analyses. The evidence suggests that targeted exercise programs with elements of both counseling and goal setting have the potential to increase the daily step activity level.

A noteworthy feature of the trials included in this review was the large variability in study interventions. The diversity in the activities prescribed (e.g., walking, strength and endurance, interventions with or without counseling) reflects the absence of consensus on the optimal activity program for cancer survivors [30]. It is likely that passive interventions such as transcutaneous muscular stimulation are not able to improve daily step activity, although they may be helpful in improving muscle strength [31], exercise capacity [32] and walking performance in laboratory settings [33].

The results of our review are comparable with other reports on programs aimed at improving walking in varied populations. There is increasing evidence that the definition of a step goal may be the key motivational factor in increasing physical activity [14]. Users of activity monitors who were given a goal, whether the 10'000 steps a day goal (as described by Tudor Locke et al.) or a personalized step goal [34], significantly increased their activity over baseline. This was not observed in studies where pedometer users were not given a goal [14].

It is important that cancer survivors are encouraged to meet the public guidelines for physical activity, because higher levels of physical activity are associated with reduced risk of overall mortality, death due to (breast) cancer, and breast cancer recurrence [35]. Recently, one study calculated that the median expected values for breast cancer survivors was 7409 steps/day, as assessed with a pedometer [15]. This is comparable with individuals with type 1 diabetes (8008 steps/day), mental retardation/intellectual disability (7787 steps/day), and HIV patients (7545 steps/day), but higher than for patients with COPD (2237 steps/day) and disabled elderly (1214 steps/day). Breast cancer patients are generally younger and less restricted in their daily physical activity than patients with many other forms of cancer [15]. Thus, not surprisingly, their median expected daily step count is higher than patients with non-small cell lung cancer (5308 steps/day) [31] and hematological cancer (5355 steps/day) [36]. Additional research is needed to determine the daily step values for patients with other forms of cancer, such as prostate, colon or head-and-neck cancer.

In order to facilitate behavior change, it is advocated that interventions be theory-based [37]. The theory of planned behavior (TPB) is a model that has been shown to predict physical activity motivation of breast cancer survivors. A combined approach using goal setting and print materials together with other TPB-based behavior change strategies (e.g. telephone counseling) may represent a promising approach [37,38]. Furthermore, social cognitive theory may be a useful framework for future investigations of physical exercise behaviour in cancer survivors [39]. This has been done in a trial of the effect of a pedometer-based telephone intervention on physical activity levels of cardiac patients who did not attend a cardiac rehabilitation program [40]. Yet, RCT's evaluating the effectiveness of the social cognitive theory in cancer survivors is warranted.

### Study limitations

We developed and utilized a structured study protocol to guide our search strategy, study selection, extraction of data and statistical analysis. However, a number of possible limitations of this review should be noted. First, the search strategy was limited to published studies identified through the selected search engines. Second, as noted, a publication bias may have been present, as well as a language bias, given that we restricted our search to English language publications. Third, as there were only 5 randomized trials, we also included several observational studies, the results of which may be affected by confounding bias due to the absence of random assignment. Fourth, many of the studies were small and may have lacked statistical power to demonstrate differences, if such differences were present. Finally, the interventions were of relatively short duration and heterogeneous in their design, and most patients investigated were breast cancer survivors.

### Future research

Despite these limitations, we believe that our review provides useful information regarding the effects of physical activity interventions aimed at improving daily step activity in cancer patients. It also provides some guidance about the components that should explicitly be considered in future interventions to enhance their effect on walking behaviour. Future studies evaluating the effects of physical activity interventions should be large RCTs carried out among diverse populations of cancer survivors. Primary outcomes for such RCTs should, as previously suggested, include both physical activity and detailed evaluation of health outcomes assessed both in the short and longer term [14].

## Conclusion

To the best of our knowledge, this is the first systematic review to include meta-analyses evaluating the effects of physical activity interventions on changes in daily walking activity in cancer survivors. Together, the studies reviewed were of relatively high methodological quality. Future studies are warranted to evaluate the effects of goal targeted physical activity, with or without counseling, on daily walking in various cancer survivor populations.

## Competing interests

The authors declare that they have no competing interests.

## Authors' contributions

RHK is the guarantor of the study. He designed the study and was the main author of the manuscript. EDB and KS designed and prepared the study protocol, assessed the methodological quality of the studies and critically revised the study and the manuscript for its content. DU initiated the study and reviewed and revised the manuscript. NKA supervised and critically reviewed and revised the study and the manuscript. All authors read and approved the final manuscript.

## Pre-publication history

The pre-publication history for this paper can be accessed here:

http://www.biomedcentral.com/1471-2407/10/406/prepub
